# Changes in family and school environment during the Covid-19 pandemic and their relationship with changes in psychological distress and loneliness among Norwegian adolescents: The HUNT study

**DOI:** 10.1016/j.ssmph.2025.101767

**Published:** 2025-02-21

**Authors:** Bodil Elisabeth Valstad Aasan, Monica Lillefjell, Věra Skalická, Steinar Krokstad, Kirsti Kvaløy, Erik R. Sund

**Affiliations:** a HUNT Research Centre, Department of Public Health and Nursing, Faculty of Medicine and Health Sciences, NTNU - Norwegian University of Science and Technology, Levanger, Norway; b Department of Neuromedicine and Movement Science, Faculty of Medicine and Health Sciences, NTNU - Norwegian University of Science and Technology, Trondheim, Norway; c Department of Public Health, Faculty of Health Sciences, UiS - University of Stavanger, Stavanger, Norway; d Department of Psychology, NTNU - Norwegian University of Science and Technology, Trondheim, Norway; e Levanger Hospital, Nord-Trøndelag Hospital Trust, Levanger, Norway; f Centre for Sami Health Research, Department of Community Medicine, UiT - The Arctic University of Norway, Tromsø, Norway

**Keywords:** Adolescents, Psychological distress, Loneliness, Family, School, Young-HUNT study, Covid-19

## Abstract

In this follow-up study, we investigated how levels of psychological distress, loneliness, family cohesion, teacher support, and peer support changed from before to during the Covid-19 pandemic among Norwegian adolescents (ages 13–19), and whether these changes were predicted by parental education. Additionally, we investigated whether changes in family cohesion, teacher support, and peer support were associated with changes in psychological distress and loneliness, and whether these change-to-change associations were moderated by parental education. Data from the Young-HUNT4 (2017–2019, T1) and Young-HUNT COVID (May–June 2021, T2) surveys were used, in which 1565 adolescents participated in both (response rate = 45%). We specified univariate and multivariate two-wave latent change score models to investigate the aims of this study. Results indicated that levels of psychological distress, loneliness, family cohesion, teacher support, and peer support worsened from T1 to T2. None of these changes were significantly predicted by parental level of education. Deteriorations in family cohesion, teacher support, and peer support were weakly related to increases in psychological distress (β = 0.17, 95% confidence interval (CI) 0.11 to 0.23; β = 0.10, 95% CI 0.04 to 0.16; β = 0.09, 95% CI 0.02 to 0.15), and loneliness (β = 0.10, 95% CI 0.05 to 0.15; β = 0.08, 95% CI 0.02 to 0.13; β = 0.12, 95% CI 0.06 to 0.18). Although largely similar, deteriorations in family cohesion were somewhat more strongly associated with increases in psychological distress among adolescents with parents of lower levels of education.

## Highlights

• Psychological distress and loneliness increased during the Covid-19 pandemic.
• Perceptions of family and school worsened during the Covid-19 pandemic.
• Parental level of education did not predict change in these measures.
• Deteriorations in family and school linked to increased distress and loneliness.
• Change in family cohesion more strongly tied to distress in low-educated families.

## 1 Introduction

Bronfenbrenner theorized that child development occurs through an increasingly complex interplay between the child and the social environment (Bronfenbrenner, 1979). Although Bronfenbrenner largely focused on early child development (Bronfenbrenner, 1979), adolescence also represents a critical period of development involving major biological, psychological, and social changes (Viner et al., 2015). Empirical studies suggest that adolescence is characterized by an increase in internalizing symptoms and loneliness (Yoon et al., 2023; von Soest, Luhmann, & Gerstorf, 2020) and that gender differences tend to widen during this time period (Yoon et al., 2023). Moreover, perceived family satisfaction, teacher support, and peer support tend to worsen during adolescence (Goldbeck et al., 2007; Wang & Dishion, 2012; Way et al., 2007).

Longitudinal studies suggest that the psychosocial environment of family and school and the mental health of adolescents could be mutually and prospectively related to each other over time (Serna et al., 2023; Urke et al., 2023; Watkins et al., 2022; Way et al., 2007). Low levels of family cohesion have been found to predict some anxiety disorders (Watkins et al., 2022), and decreases in teacher and peer support have been found to be related to increases in depressive symptoms (Way et al., 2007). However, adolescent mental health problems have also been found to be predictive of parent-reported changes in family functionality (Serna et al., 2023) and self-reported school climate (Urke et al., 2023).

Socioeconomic inequalities in health are found in most countries (Mackenbach et al., 2008), and studies suggest that socioeconomic inequalities in mental health and loneliness emerge in childhood and adolescence (Qualter et al., 2021; Reiss, 2013). Although the social conditions in which we live our lives, known as the social determinants of health, are considered to influence the health and well-being of everyone, the unequal distribution of health determinants is considered an underlying cause of health inequalities (CSDH, 2008). Empirical studies suggest that socioeconomic health inequalities are partially explained by material, psychosocial, and behavioral factors in particular (Moor et al., 2017).

In 2020, a natural experiment occurred when the Covid-19 pandemic and the subsequent restrictions impacted every level of society in many countries, thus providing a unique opportunity to investigate potential societal and health-related impacts. In Norway, a national lockdown was implemented on March 12, 2020, resulting in digital schooling and minimal physical contact with others for extended periods of time (Helsedirektoratet, 2020).

Some studies indicate that Norwegian adolescents were negatively impacted by the Covid-19 pandemic, as adolescents during the pandemic reported lower quality of life than pre-pandemic European norms (Lehmann et al., 2023). Moreover, in an investigation of trends in psychosocial well-being before and one year into the Covid-19 pandemic, one study found a disproportionate increase in depressive symptoms and a disproportionate decrease in optimism about the future during the Covid-19 pandemic compared to relative trends (von Soest et al., 2022). However, peer relationships, parental relationships, and loneliness were not found to drastically change during the Covid-19 pandemic (von Soest et al., 2022).

On the other hand, a few studies also suggest that the mental health of Norwegian adolescents did not drastically worsen during the pandemic (Burdzovic Andreas & Brunborg, 2021; Hafstad et al., 2021). While one study concluded that the observed increases in anxiety and depressive symptoms were age-related rather than related to the pandemic (Hafstad et al., 2021), another study observed no meaningful differences when comparing an adolescent cohort before and during the pandemic in terms of reported physical health, depressive symptoms, and number of friends (Burdzovic Andreas & Brunborg, 2021). Moreover, in another study, some adolescents reported that parts of their lives improved during the national lockdown (Lehmann et al., 2021).

The Covid-19 pandemic may, however, have had a differential impact on various socioeconomic groups (von Soest et al., 2022). Studies conducted during the first wave of the pandemic produced somewhat contrasting results: one study found that adolescents from less affluent families reported more adversity related to their living conditions (Lehmann et al., 2021), a second study found diminishing socioeconomic inequalities in life satisfaction (von Soest, Bakken, et al., 2020), and a third study observed mostly stable socioeconomic inequalities in depressive symptoms, loneliness, life satisfaction, and quality of life (Myhr et al., 2021). However, when assessing psychosocial well-being one year into the Covid-19 pandemic, one study found that adolescents from parents with low levels of education were disproportionately impacted by the pandemic (von Soest et al., 2022). In particular, compared with adolescents from parents with higher levels of education, those from parents with low levels of education showed a greater decrease in peer relationships, parental relationships, and optimism about the future, along with a greater increase in depressive symptoms and loneliness (von Soest et al., 2022).

Although the Covid-19 pandemic drastically changed people's lives, empirical studies provide ambiguous results regarding the extent to which the Covid-19 pandemic impacted the lives and well-being of adolescents. Moreover, we still lack evidence pertaining to the consequences of the Covid-19 pandemic and the potential differential consequences of changes in the social environment of family and school on changes in adolescent mental health. As such, the overall aim of the present study is to investigate changes in family and school environment and their relationship with changes in psychological distress and loneliness among adolescents before and during the Covid-19 pandemic. Specifically, we investigate the following research questions:

1. How do adolescent psychological distress, loneliness, family cohesion, teacher support, and peer support change during the Covid-19 pandemic compared to the pre-pandemic period, and are these changes predicted by parental education level?
2. How are changes in family cohesion, teacher support, and peer support related to changes in adolescent psychological distress and loneliness, and do these change-to-change associations vary according to parental education level?

## 2 Methods

### 2.1 Sample and study procedure

The present study used data from the Young-HUNT4 and Young-HUNT COVID surveys (*n* = 1565). In both surveys, a probability sampling method was used, although the inclusion criteria were somewhat different. In the Young-HUNT4 survey, all adolescents (ages 13–19) residing in the former county of Nord-Trøndelag were invited to participate (Holmen et al., 2014; Rangul et al., 2024). The survey was conducted in 2017–2019 (T1) and data were collected during school hours. In total, 10 608 adolescents were invited and 8099 participated (response rate = 76%) (Rangul et al., 2024). The Young-HUNT COVID survey, a follow-up study initiated in response to the Covid-19 pandemic, was conducted in May–June 2021 (T2). The inclusion criteria for this study were participation in the Young-HUNT4 survey and being at least 16 years old at the time the Young-HUNT COVID survey was conducted. Due to ongoing school lockdowns and teacher strikes, the questionnaire was made available online so that adolescents could participate during their leisure time. In total, 3446 adolescents were invited and 1565 participated (response rate = 45%), and all included measures were assessed at both time points.

### 2.2 Measures

#### 2.2.1 Family cohesion

Perceived family cohesion was measured using three items from the Resilience Scale for Adolescents (READ; Hjemdal, Friborg, Stiles, Martinussen, & Rosenvinge, 2006; von Soest et al., 2010). The questions were as follows: “In my family we share views of what is important in life,” “I feel comfortable with my family,” and “My family views the future as positive, even when very sad things happen.” Thinking about the last month, the adolescents reported to what extent they agreed with each statement on a 5-point Likert scale (1 = *Totally agree*, 2 = *Agree,* 3 = *Average,* 4 = *Disagree,* 5 = *Totally disagree*), with higher scores indicating lower levels of family cohesion. Cronbach's alpha was 0.76 (T1) and 0.87 (T2).

#### 2.2.2 Teacher and peer support

Perceived teacher support and peer support were measured using seven questions adapted from the Health Behavior in School-Aged Children (HBSC) survey (Currie et al., 2000). In accordance with previous studies using similar measures which distinguish between teacher and classmate support (Torsheim et al., 2000), we used the following four questions to measure teacher support: “My main teacher treats me with respect,” “I receive help from my teacher when I need it,” “When I have problems or I'm sad I can talk to my teacher,” and “My teacher encourages good social environment and friendship in the class.” The following three questions measured peer support: “My fellow pupils treat me with respect,” “If someone in class are treated poorly or unfairly we help each other,” and “I've become friends with many in this class.” All items were answered on a 4-point Likert scale (1 = *Strongly agree*, 2 = *Agree*, 3 = *Disagree*, 4 = *Strongly disagree*), with higher scores indicating lower levels of teacher and peer support. Cronbach's alpha was 0.83 (T1) and 0.86 (T2) for teacher support, and 0.74 (T1) and 0.78 (T2) for peer support, indicating acceptable internal consistency.

#### 2.2.3 Psychological distress

Psychological distress was measured using the Symptom Checklist-5 (SCL-5; Strand et al., 2003), a shortened version of the Hopkins Symptom Checklist (HSCL; Derogatis, Lipman, Rickels, Uhlenhuth, & Covi, 1974). The adolescents were asked to report the degree to which they had experienced the following five symptoms of depression and anxiety in the last two weeks: “Felt afraid and anxious?”, “Felt tense or uneasy?”, “Felt hopelessness when you think of the future?”, “Felt dejected or sad?”, and “Worried too much about various things?”. All items were answered on a 4-point scale (1 = *Not bothered*, 2 = *A little bothered*, 3 = *Quite bothered*, 4 = *Very bothered*), with higher scores indicating greater levels of psychological distress. Cronbach's alpha was 0.86 (T1) and 0.87 (T2).

#### 2.2.4 Loneliness

Loneliness was assessed using two questions adapted from the Three-Item Loneliness Scale (Hughes et al., 2004). Thinking about school and spare time, the adolescents were asked to rate how often they feel: “That you are lonely,” and “That you miss socializing with others.” Both questions were answered on a 5-point scale (1 = *Very rarely or never*, 2 = *Rarely*, 3 = *Sometimes*, 4 = *Often*, 5 = *Very often*), with higher scores indicating greater levels of loneliness. Cronbach's alpha was 0.79 (T1) and 0.85 (T2).

#### 2.2.5 Parental education level

Parental education level was used as a proxy measure of socioeconomic position and was obtained from Statistics Norway. To distinguish between adolescents with high and low parental education, we defined the group of high parental education as at least one parent having completed a higher education. We also obtained a family ID for each participant to identify adolescents belonging to the same family.

### 2.3 Statistical analyses

Descriptive statistics were conducted for all variables at T1 and T2. We also compared participants and non-participants at baseline using chi-square tests for categorical variables and Mann–Whitney U tests for continuous variables to investigate the representativeness of the sample. These preliminary analyses were conducted using Stata version 18 (StataCorp, 2023).

To investigate changes in psychological distress, loneliness, family cohesion, teacher support, and peer support, we specified separate univariate two-wave latent change score (2W-LCS) models (Henk & Castro-Schilo, 2016). The latent change scores were created by forming latent variables at T1 and T2 following the criteria of strong measurement invariance, allowing each separate item residual at T1 and T2 to covary (Henk & Castro-Schilo, 2016). Thus, the latent change score represents intra-individual change, and the mean latent change score represents the average group change. All constructs were coded such that a higher score indicated a *poorer* outcome. Thus, a statistically significant *positive* latent change score, indicating an increase, could be interpreted as a deterioration while a significant *negative* latent change score, indicating a decrease, could be interpreted as an improvement from T1 to T2 for all constructs. Moreover, a statistically significant variance indicated substantial individual heterogeneity in this mean group change.

These univariate 2W-LCS models were then used to investigate whether change in adolescent psychological distress, loneliness, family cohesion, teacher support, and peer support was predicted by parental education by regressing the latent change scores on parental education (low = 0, high = 1). Parental education, sex (female = 0, male = 1), and age (rounded to the nearest integer) were included as covariates for the latent variables at T1, and sex was included as a covariate for the latent change scores.

**Fig. 1:**
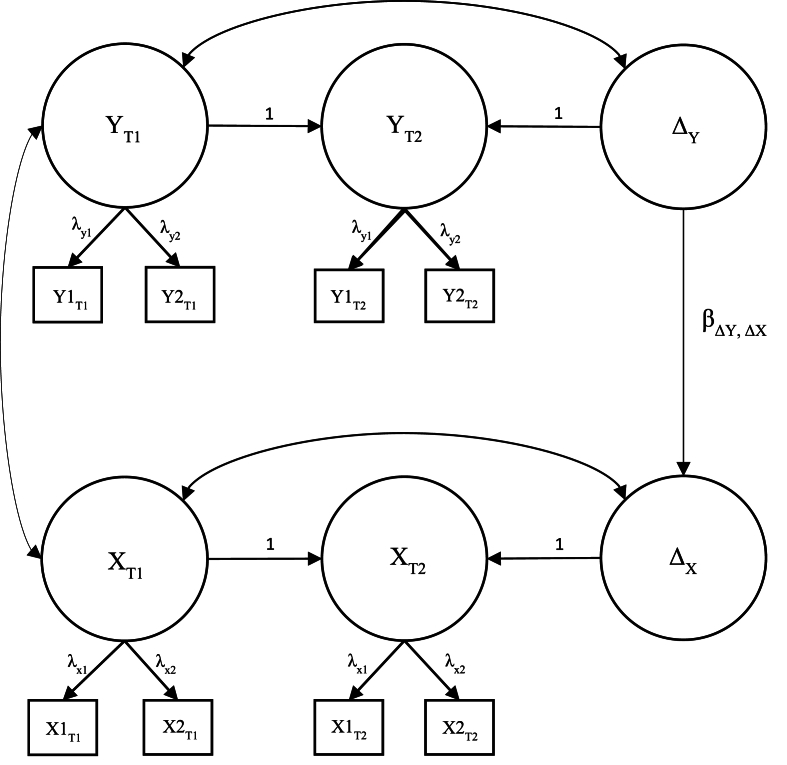
A simplified path diagram depicting the base model of the two-wave latent change score model. Deltas represent latent change scores. Y represents family cohesion, teacher support, or peer support, while X represents psychological distress or loneliness.

To investigate whether changes in family cohesion, teacher support, and peer support were associated with changes in adolescent psychological distress and loneliness, we used multivariate 2W-LCS models (Henk & Castro-Schilo, 2016). We created six separate 2W-LCS models by combining the univariate 2W-LCS models of family cohesion, teacher support, and peer support with the univariate 2W-LCS models of psychological distress and loneliness (see Fig. 1 for path diagram). We regressed the latent change score of psychological distress and loneliness on the latent change score of family cohesion, teacher support, and peer support to investigate change-to-change associations. We included age, sex, and parental education as covariates for the latent variables at T1, and sex was included as a covariate for the latent change scores. Lastly, we investigated whether the change-to-change associations varied between low and high parental education using multi-group analyses. Age and sex were included as covariates as before, and we compared the models with the constrained and unconstrained change-to-change associations using Satorra-Bentler scaled chi-square tests (Satorra & Bentler, 2001). Both the univariate and multivariate 2W-LCS models were constructed using Mplus version 8.10 (Muthén & Muthén, 2017). Since the proportion of missing data was low (≤5% for each item), the risk of biased estimates was considered low. Assuming the data were missing at random (MAR), we employed full information maximum likelihood (FIML) estimation, which has been found to be effective in handling missing data under this assumption (Enders & Bandalos, 2001). We handled non-normality by employing maximum likelihood estimation with robust standard errors (MLR; Muthén & Muthén, 2017). Moreover, we handled potential clustering within families by employing type = complex using family ID as the cluster ID (Muthén & Muthén, 2017). Model fit was evaluated using the recommendations by Hu and Bentler (1998) and is reported in a supplementary file.

## 3 Results

### 3.1 Sample characteristics

The study sample included 1565 adolescents (61% girls); full descriptive information is reported in Table 1. Results from the chi-square tests showed that a higher percentage of girls (χ^2^ (1) = 69.64, *p* < 0.001) and a higher percentage of adolescents from highly educated parents (χ^2^ (1) = 14.62, *p* < 0.001) were included in the study sample compared to non-participants. According to the Mann–Whitney U tests, the mean levels of psychological distress (z = −1.80, *p* = 0.07), loneliness (z = −0.89, *p* = 0.37), family cohesion (z = 0.55, *p* = 0.58), teacher support (z = −0.15, *p* = 0.88), and peer support (z = −0.46, *p* = 0.64) did not vary considerably between participants and non-participants. However, the study sample was younger than non-participants (z = 6.09, *p* < 0.001).

**Table 1:** Descriptive statistics of study sample (*N* = 1565).

	T1			T2		
	M/N	SD/%	Mis. %	M/N	SD/%	Mis. %
Sex						
Girls	960	61%				
Boys	605	39%				
Age	14.68	1.08		17.86	0.91	
Parental education						
Low	502	33%				
High	1015	67%	3%			
Psychological distress						
Anxiety	1.48	0.74	2%	1.59	0.81	2%
Tense	1.51	0.74	3%	1.78	0.89	2%
Hopeless	1.53	0.84	3%	1.86	0.99	2%
Sad	1.81	0.92	2%	2.02	0.92	2%
Worry	1.88	0.97	3%	2.13	1.04	2%
Loneliness						
Socializing	1.99	1.14	3%	2.32	1.26	2%
Lonely	1.80	1.11	3%	2.19	1.25	2%
Teacher support						
Respect	1.47	0.67	3%	1.54	0.74	3%
Help	1.64	0.66	3%	1.79	0.72	3%
Support	2.12	0.92	3%	2.22	0.97	3%
Cohesion	1.74	0.74	4%	1.85	0.81	3%
Peer support						
Respect	1.63	0.68	3%	1.66	0.71	3%
Help each other	1.88	0.72	5%	2.01	0.79	4%
Friends	1.60	0.76	3%	1.79	0.85	3%
Family cohesion						
Important	2.17	1.09	3%	2.37	1.14	3%
Comfortable	1.32	0.73	2%	1.66	1.05	2%
Positive	1.56	0.91	3%	1.86	1.08	3%

*Note.* Higher scores on psychological distress, loneliness, family cohesion.

Teacher support, and peer support indicate a poorer outcome.

M, mean; N, number; SD, standard deviation; Mis., missing.

### 3.2 Univariate 2W-LCS models

Reporting standardized estimates, results from the unadjusted univariate 2W-LCS models showed a statistically significant positive latent change score for all constructs, indicating that on average, the levels of psychological distress (μΔ = 0.34, 95% confidence interval [CI] 0.28 to 0.39), loneliness (μΔ = 0.32, 95% CI 0.26 to 0.37), family cohesion (μΔ = 0.32, 95% CI 0.27 to 0.36), teacher support (μΔ = 0.15, 95% CI 0.10 to 0.21), and peer support (μΔ = 0.18, 95% CI 0.12 to 0.25) worsened from T1 to T2. A statistically significant variance in the estimated latent change scores indicates substantial heterogeneity around the mean group change. Furthermore, parental education was not found to predict any of the latent change scores (Table 2).

**Table 2:** Univariate 2W-LCS models predicting change.

Parental education→	Estimate	SE	95% CI
ΔPsychological distress	−0.02	0.03	−0.07 to 0.04
ΔLoneliness	0.03	0.03	−0.03 to 0.08
ΔFamily cohesion	−0.01	0.03	−0.06 to 0.05
ΔTeacher support	−0.06	0.03	−0.11 to 0.00
ΔPeer support	0.00	0.03	−0.06 to 0.06

*Note.* Latent variables at T1 were adjusted for sex, age, and parental education level. Latent change scores were adjusted for sex.

2W-LCS, two-wave latent change score; SE, standard error; CI, confidence interval; Δ, latent change score.

### 3.3 Multivariate 2W-LCS models

Results from the multivariate 2W-LCS models are presented in Table 3. Since all latent change scores indicated a deterioration from T1 to T2, the results suggest that deteriorations in family cohesion were weakly related to increases in psychological distress (β = 0.17, 95% CI 0.11 to 0.23) and loneliness (β = 0.10, 95% CI 0.05 to 0.15). Similarly, deteriorations in teacher support were weakly related to increases in psychological distress (β = 0.10, 95% CI 0.04 to 0.16) and loneliness (β = 0.08, 95% CI 0.02 to 0.13). Lastly, deteriorations in peer support were weakly related to increases in psychological distress (β = 0.09, 95% CI 0.02 to 0.15) and loneliness (β = 0.12, 95% CI 0.06 to 0.18).

**Table 3:** Standardized estimates from the multivariate 2W-LCS models.

	ΔPsychological distress			ΔLoneliness		
	Estimate	SE	95% CI	Estimate	SE	95% CI
Family cohesion						
ΔFamily cohesion→	0.17	0.03	0.11 to 0.23	0.10	0.03	0.05 to 0.15
Intercepts						
ΔFamily cohesion	0.26	0.04	0.19 to 0.33	0.26	0.04	0.19 to 0.33
ΔOutcome	0.39	0.04	0.31 to 0.46	0.37	0.04	0.29 to 0.44
Residual variances						
ΔFamily cohesion	1.00	0.00	1.00 to 1.00	1.00	0.00	1.00 to 1.00
ΔOutcome	0.96	0.01	0.93 to 0.98	0.98	0.01	0.97 to 1.00
Teacher support						
ΔTeacher support→	0.10	0.03	0.04 to 0.16	0.08	0.03	0.02 to 0.13
Intercepts						
ΔTeacher support	0.09	0.04	0.02 to 0.16	0.09	0.04	0.02 to 0.16
ΔOutcome	0.42	0.04	0.34 to 0.50	0.39	0.04	0.32 to 0.47
Residual variances						
ΔTeacher support	0.99	0.01	0.99 to 1.00	0.99	0.00	0.99 to 1.00
ΔOutcome	0.98	0.01	0.96 to 0.99	0.99	0.01	0.97 to 1.00
Peer support						
ΔPeer support→	0.09	0.03	0.02 to 0.14	0.12	0.03	0.06 to 0.18
Intercepts						
ΔPeer support	0.15	0.04	0.07 to 0.22	0.15	0.04	0.07 to 0.23
ΔOutcome	0.42	0.04	0.34 to 0.49	0.38	0.04	0.31 to 0.46
Residual variances						
ΔPeer support	1.00	0.00	0.99 to 1.00	1.00	0.00	0.99 to 1.00
ΔOutcome	0.98	0.01	0.96 to 0.99	0.98	0.01	0.96 to 0.99

*Note*. Latent variables at T1 were adjusted for sex, age, and parental education level. Latent change scores were adjusted for sex.

2W-LCS, two-wave latent change score; SE, standard error; CI, confidence interval; Δ, latent change score.

The multi-group analyses indicated that most of these change-to-change associations were not moderated by parental education. However, the association between change in family cohesion and change in psychological distress seemed to be moderated by parental education (χ^2^ (1) = 4.85, *p* = 0.03) showing a somewhat stronger change-to-change association for low parental education (β = 0.26, 95% CI 0.16 to 0.35) than high parental education (β = 0.14, 95% CI 0.07 to 0.21).

## 4 Discussion

This study shows that Norwegian adolescents’ psychological distress, loneliness, family cohesion, teacher support, and peer support tended to worsen from before to during the Covid-19 pandemic. These changes were not predicted by parental education. In addition, deteriorations in family cohesion, teacher support, and peer support were weakly associated with increases in psychological distress and loneliness. These change-to-change associations were mostly similar between high and low parental education, although deteriorations in family cohesion showed a stronger association with increases in psychological distress among adolescents from parents with low levels of education.

The present findings are in accordance with previous studies showing increases in symptoms of depression and anxiety among Norwegian adolescents (Hafstad et al., 2021) and decreases in family cohesion and social resources among Swiss adolescents during the pandemic (Janousch et al., 2022). In contrast, another study of Norwegian adolescents reported that psychological well-being decreased, perceived autonomy and parent relations remained stable, and perceived social and peer support increased during the pandemic (Lehmann et al., 2023). It should be noted, however, that the present study compared measurements made before and during the Covid-19 pandemic while this other study compared measurements made early in the pandemic and later in the pandemic (Lehmann et al., 2023). Comparing these patterns of change may therefore be problematic, as adolescents may have been more impacted early in the pandemic when the national lockdown was at its strictest compared to when the national lockdown gradually lessened. As Lehmann et al. (2023) point out, improvement in perceived social and peer support may have been due to greater opportunity for face-to-face contact during the second measurement than the first.

In general, it may be difficult to conclude whether the pattern of change observed in the present study is a result of the pandemic or is related to other factors. On the one hand, two studies reported that French and Norwegian adolescents had poorer physical and psychological well-being compared with European pre-pandemic data (Bourion-Bédès et al., 2022; Lehmann et al., 2023), suggesting a potential negative impact of the Covid-19 pandemic. However, another study suggested that Norwegian adolescents were not significantly impacted by the Covid-19 pandemic, as no differences were observed in self-reported physical health, depressive symptoms, or the number of friends when comparing an adolescent cohort from before the pandemic with one from during the pandemic (Burdzovic Andreas & Brunborg, 2021). Moreover, another study suggested that increases in symptoms of depression and anxiety among Norwegian adolescents during the Covid-19 pandemic were related to increases in age, as the observed changes were minimal and became statistically non-significant when age was included as a covariate (Hafstad et al., 2021). Hence, the pattern of changes observed in the present study may reflect a broader developmental trend occurring during adolescence, in accordance with studies showing that loneliness and mental health problems tend to increase (von Soest, Luhmann, & Gerstorf, 2020; Yoon et al., 2023), and perceptions of family and school tend to worsen during adolescence (Wang & Dishion, 2012; Way et al., 2007).

Changes in psychological distress, loneliness, family cohesion, teacher support, and peer support were not predicted by parental education. Focusing on studies conducted during the Covid-19 period, the present results align with one study which found that socioeconomic inequalities in depressive symptoms, life satisfaction, loneliness, and quality of life among Norwegian adolescents did not significantly change during the Covid-19 pandemic (Myhr et al., 2021). However, a nationwide study conducted one year into the pandemic found that adolescents with lower parental education exhibited greater increases in loneliness and depressive symptoms, along with greater decreases in perceived peer relationships and parental relationships, compared with adolescents with higher parental education (von Soest et al., 2022). A potential explanation for these contrasting findings may be regional differences, as it is possible that the Covid-19 pandemic may have affected regions differently. However, when investigating potential differences across Norwegian municipalities, von Soest et al. (2022) found that neither infection rates nor restrictions were associated with changes in psychosocial well-being among adolescents. Nevertheless, regional differences may exist regarding the potential differential impact of the Covid-19 pandemic across socioeconomic backgrounds.

The longitudinal relationship between the mental well-being of children and the social environment of family and school is likely to involve a complex reciprocal process occurring over time (Bronfenbrenner, 1979). This is reflected in the fact that some empirical studies suggest that the psychosocial family environment predicts adolescent mental health over time (Watkins et al., 2022) while others suggest that adolescent mental health predicts family functionality over time (Serna et al., 2023). Similarly, one study of middle-school students in the US found support for a unidirectional relationship between decreases in teacher support and increases in depressive symptoms and a bidirectional relationship between decreases in peer support and increases in depressive symptoms (Way et al., 2007). Moreover, a Norwegian study concluded that the mental well-being of high school students was predictive of changes in perceived school climate and not vice versa (Urke et al., 2023). Hence, it is not clear how the present findings should be interpreted, as the weak change-to-change associations could reflect either a unidirectional or bidirectional relationship.

Deteriorations in teacher and peer support were both found to be similarly associated with increases in loneliness and psychological distress among adolescents from parents with low and high levels of education. These findings align with previous studies suggesting that the longitudinal relationship between school belongingness and adolescent mental health is similar across household income (Vaz et al., 2014). Similarly, another study conducted during the Covid-19 pandemic found that household income did not moderate the effect of remote learning on the feelings of stress and the positive and negative affect among adolescents from the US (Wang, Scanlon, et al., 2023). The present findings may therefore indicate that the school environment is similarly related to the mental health and loneliness of adolescents irrespective of their socioeconomic background.

We also found no evidence for a moderating effect of parental education on the change-to-change association between family cohesion and loneliness. However, the change-to-change association between family cohesion and psychological distress was moderated by parental education, as deteriorations in family cohesion showed a stronger association with increases in psychological distress among adolescents from parents with low levels of education compared with high levels of education. This finding is in contrast with the results from one study which reported no differential impact of parental support, peer support, and parent-child conflict on the psychological well-being of adolescents across income groups during the Covid-19 pandemic (Wang, Henry, et al., 2023). However, the same study also reported that food insecurity and daily health-related stress were stronger risk factors for negative affect among low-income families than high-income families (Wang, Henry, et al., 2023), which aligns with the present results.

It may sound counterintuitive that parental education level exerted a moderating effect on the change-to-change association between family cohesion and psychological distress, yet it was not a significant predictor of changes in either family cohesion or psychological distress. However, because health determinants seem to follow a social gradient (CSDH, 2008), the significance of these health determinants may vary across different socioeconomic contexts as well (Diderichsen, Evans, & Whitehead, 2001). For example, previous studies have shown that adolescents from lower socioeconomic backgrounds are more likely to lack both social resources, family resources, and personal resources compared with adolescents from higher socioeconomic backgrounds (Schmidtke et al., 2021). This unequal distribution of psychosocial resources may, in addition to being a risk factor in itself, also result in differential vulnerability across different socioeconomic contexts (Diderichsen et al., 2001). The present findings may therefore reflect a differential vulnerability, as changes in family cohesion could be more strongly related to changes in psychological distress among adolescents from lower socioeconomic backgrounds due to the lack of important compensatory resources outside the family context. Moreover, the circumstances of the Covid-19 pandemic may have amplified this differential vulnerability, as the availability of important compensatory psychosocial resources (e.g., school and friends) was impacted, which could have had different consequences across socioeconomic contexts.

### 4.1 Strengths and limitations

Two strengths of the present study are the relatively large sample size and the use of recent methodological advancements, allowing us to investigate intra-individual change and change-to-change associations using two-occasion data (Henk & Castro-Schilo, 2016). However, two-occasion data still present a limitation, as we cannot assess longitudinal trends or disentangle temporal precedence in the explored changes (Henk & Castro-Schilo, 2016). Additionally, having only two measurements may limit our ability to fully assess a potential moderating effect of parental education level. As such, it will be important to investigate the present research questions using longitudinal data with more time points.

Another limitation is the low response rate, likely due to ongoing school lockdowns and a teacher strike during data collection, which limited participation during school hours. Although the baseline levels of psychological distress, loneliness, family cohesion, teacher support, and peer support did not differ significantly between participants and non-participants, it is possible that changes in these dimensions could vary between these groups. Additionally, the underrepresentation of boys, older adolescents, and adolescents from parents with lower levels of education may limit the representativeness of the sample. This underrepresentation could introduce bias in the estimates of change and in the predictive value of parental education level.

Lastly, the present study includes data from before and during the Covid-19 pandemic, which is considered a strength as it allows for investigating changes prior to and during the pandemic. However, we cannot conclude whether the findings are solely due to the circumstances of the Covid-19 pandemic or are influenced by other factors. Specially, it is important to acknowledge that adolescence itself involves a myriad of social and psychological changes. As such, it is possible that the present findings represent more general changes occurring during adolescence rather than changes due to the Covid-19 pandemic. Future studies are therefore needed to investigate the generalizability of the present findings by examining how changes in the family and school environment relate to changes in adolescent mental health, and how these longitudinal relationships may vary across socioeconomic backgrounds in a context unaffected by the Covid-19 pandemic.

### 4.2 Conclusions

Although there seemed to be substantial individual variation around the investigated latent change scores, there was a clear group tendency suggesting that family cohesion, teacher support, peer support, psychological distress, and loneliness worsened from before to during the Covid-19 pandemic. Moreover, deteriorations in family cohesion, teacher support, and peer support were weakly associated with increases in psychological distress and loneliness. Although we were unable to investigate the temporality of these change-to-change associations, our findings suggest that programs aimed towards supporting families and schools may be valuable in preventing the often-seen worsening of mental health problems and loneliness during adolescence. These initiatives may be especially beneficial for adolescents from parents with low levels of education.

## CRediT authorship contribution statement

**Bodil Elisabeth Valstad Aasan:** Writing – original draft, Methodology, Formal analysis, Conceptualization. **Monica Lillefjell:** Writing – review & editing, Supervision, Project administration, Funding acquisition, Conceptualization. **Věra Skalická:** Writing – review & editing, Methodology, Formal analysis. **Steinar Krokstad:** Writing – review & editing, Supervision, Conceptualization. **Kirsti Kvaløy:** Writing – review & editing, Conceptualization. **Erik R. Sund:** Writing – review & editing, Supervision, Methodology, Conceptualization.

## Ethical statement

The study was approved by the Regional Committee for Medical and Health Research Ethics (REK-Midt ref. 262408).

## Declaration of interest

The authors declare that they have no known competing financial interests or personal relationships that could have appeared to influence the work reported in this paper.

## Data Availability

The authors do not have permission to share data, but access to HUNT data can be requested via https://hunt-db.medisin.ntnu.no/hunt-db/variablelist.

## References

[bib1] Bourion-Bédès S., Rousseau H., Batt M., Tarquinio P., Lebreuilly R., Sorsana C. (2022). The effects of living and learning conditions on the health-related quality of life of children and adolescents during the COVID-19 lockdown in the French Grand Est region. BMC Public Health.

[bib2] Bronfenbrenner U. (1979).

[bib3] Burdzovic Andreas J., Brunborg G.S. (2021). Self-reported mental and physical health among Norwegian adolescents before and during the COVID-19 pandemic. JAMA Network Open.

[bib4] CSDH (2008). Closing the gap in a generation: Health equity through action on the social determinants of health: Final report of the commission on social determinants of health. https://www.who.int/publications/i/item/WHO-IER-CSDH-08.1.

[bib5] Currie C., Hurrelmann K., Settertobulte W., Smith B., Todd J. (2000).

[bib6] Derogatis L.R., Lipman R.S., Rickels K., Uhlenhuth E.H., Covi L. (1974). The Hopkins symptom checklist (HSCL): A self-report symptom inventory. Behavioral Science.

[bib7] Diderichsen F., Evans T., Whitehead M., Evans T., Whitehead M., Diderichsen F., Bhuiya A., Wirth M. (2001). Challenging inequities in health: From ethics to action.

[bib8] Enders C., Bandalos D. (2001). The relative performance of full information maximum likelihood estimation for missing data in structural equation models. Structural Equation Modeling: A Multidisciplinary Journal.

[bib10] Goldbeck L., Schmitz T.G., Besier T., Herschbach P., Henrich G. (2007). Life satisfaction decreases during adolescence. Quality of Life Research.

[bib11] Hafstad G.S., Sætren S.S., Wentzel-Larsen T., Augusti E.M. (2021). Adolescents' symptoms of anxiety and depression before and during the Covid-19 outbreak—a prospective population-based study of teenagers in Norway. The Lancet Regional Health Europe.

[bib12] Helsedirektoratet Helsedirektoratets anbefalinger om håndtering av covid-19 per 24. Mars. https://www.helsedirektoratet.no.

[bib13] Henk C.M., Castro-Schilo L. (2016). Preliminary detection of relations among dynamic processes with two-occasion data. Structural Equation Modeling: A Multidisciplinary Journal.

[bib14] Hjemdal O., Friborg O., Stiles T.C., Martinussen M., Rosenvinge J.H. (2006). A new scale for adolescent resilience: Grasping the central protective resources behind healthy development. Measurement and Evaluation in Counseling and Development.

[bib15] Holmen T.L., Bratberg G., Krokstad S., Langhammer A., Hveem K., Midthjell K. (2014). Cohort profile of the young-HUNT study, Norway: A population-based study of adolescents. International Journal of Epidemiology.

[bib16] Hu L., Bentler P.M. (1998). Fit indices in covariance structure modeling: Sensitivity to underparameterized model misspecification. Psychological Methods.

[bib17] Hughes M.E., Waite L.J., Hawkley L.C., Cacioppo J.T. (2004). A short scale for measuring loneliness in large surveys: Results from two population-based studies. Research on Aging.

[bib18] Janousch C., Anyan F., Morote R., Hjemdal O. (2022). Resilience patterns of Swiss adolescents before and during the COVID-19 pandemic: A latent transition analysis. International Journal of Adolescence and Youth.

[bib19] Lehmann S., Haug E., Bjørknes R., Mjeldheim Sandal G., T Fadnes L., Skogen J.C. (2023). Quality of life among young people in Norway during the COVID-19 pandemic. A longitudinal study. European Child & Adolescent Psychiatry.

[bib20] Lehmann S., Skogen J.C., Haug E., Mæland S., Fadnes L.T., Sandal G.M. (2021). Perceived consequences and worries among youth in Norway during the COVID-19 pandemic lockdown. Scandinavian Journal of Public Health.

[bib21] Mackenbach J.P., Stirbu I., Roskam A.-J.R., Schaap M.M., Menvielle G., Leinsalu M. (2008). Socioeconomic inequalities in health in 22 European countries. New England Journal of Medicine.

[bib22] Moor I., Spallek J., Richter M. (2017). Explaining socioeconomic inequalities in self-rated health: A systematic review of the relative contribution of material, psychosocial and behavioural factors. Journal of Epidemiology & Community Health.

[bib23] Muthén L.K., Muthén B.O. (2017). Mplus User’s Guide.

[bib24] Myhr A., Naper L.R., Samarawickrema I., Vesterbekkmo R.K. (2021). Impact of COVID-19 pandemic lockdown on mental well-being of Norwegian adolescents during the first wave-socioeconomic position and gender differences. Frontiers in Public Health.

[bib25] Qualter P., Hennessey A., Yang K., Chester K.L., Klemera E., Brooks F. (2021). Prevalence and social inequality in youth loneliness in the UK. International Journal of Environmental Research and Public Health.

[bib26] Rangul V., Holmen T.L., Langhammer A., Ingul J.M., Pape K., Fenstad J.S. (2024). Cohort profile update: The Young-HUNT study, Norway. International Journal of Epidemiology.

[bib27] Reiss F. (2013). Socioeconomic inequalities and mental health problems in children and adolescents: A systematic review. Social Science & Medicine.

[bib28] Satorra A., Bentler P.M. (2001). A scaled difference chi-square test statistic for moment structure analysis. Psychometrika.

[bib29] Schmidtke C., Geene R., Hölling H., Lampert T. (2021). Mental health issues in childhood and adolescence, psychosocial resources and socioeconomic status—an analysis of the KiGGS Wave 2 data. Journal of Health Monitoring.

[bib30] Serna A., Thakur H., Cohen J.R., Briley D.A. (2023). Testing the temporal precedence of family functioning and child psychopathology in the LONGSCAN sample. Development and Psychopathology.

[bib31] StataCorp (2023).

[bib32] Strand B.H., Dalgard O.S., Tambs K., Rognerud M. (2003). Measuring the mental health status of the Norwegian population: A comparison of the instruments SCL-25, SCL-10, SCL-5 and MHI-5 (SF-36). Nordic Journal of Psychiatry.

[bib33] Torsheim T., Wold B., Samdal O. (2000). The teacher and classmate support scale: Factor structure, test-retest reliability and validity in samples of 13-and 15-year-old adolescents. School Psychology International.

[bib34] Urke H.B., Kristensen S.M., Bøe T., Gaspar De Matos M., Wiium N., Årdal E. (2023). Perceptions of a caring school climate and mental well-being: A one-way street? Results from a random intercept cross-lagged panel model. Applied Developmental Science.

[bib35] Vaz S., Falkmer M., Parsons R., Passmore A.E., Parkin T., Falkmer T. (2014). School belongingness and mental health functioning across the primary-secondary transition in a mainstream sample: Multi-group cross-lagged analyses. PLoS One.

[bib36] Viner R.M., Ross D., Hardy R., Kuh D., Power C., Johnson A. (2015). Life course epidemiology: Recognising the importance of adolescence. Journal of Epidemiology & Community Health.

[bib37] von Soest T., Bakken A., Pedersen W., Sletten M.A. (2020). Life satisfaction among adolescents before and during the COVID-19 pandemic. Tidsskrift for Den norske legeforening.

[bib38] von Soest T., Kozák M., Rodríguez-Cano R., Fluit D.H., Cortés-García L., Ulset V.S. (2022). Adolescents' psychosocial well-being one year after the outbreak of the COVID-19 pandemic in Norway. Nature Human Behaviour.

[bib39] von Soest T., Luhmann M., Gerstorf D. (2020). The development of loneliness through adolescence and young adulthood: Its nature, correlates, and midlife outcomes. Developmental Psychology.

[bib40] von Soest T., Mossige S., Stefansen K., Hjemdal O. (2010). A validation study of the resilience scale for adolescents (READ). Journal of Psychopathology and Behavioral Assessment.

[bib41] Wang M.-T., Dishion T.J. (2012). The trajectories of adolescents' perceptions of school climate, deviant peer affiliation, and behavioral problems during the middle school years. Journal of Research on Adolescence.

[bib42] Wang M.-T., Henry D.A., Scanlon C.L., Del Toro J., Voltin S.E. (2023). Adolescent psychosocial adjustment during COVID-19: An intensive longitudinal study. Journal of Clinical Child and Adolescent Psychology.

[bib43] Wang M.-T., Scanlon C.L., Del Toro J., Qin X. (2023). Adolescent psychological adjustment and social supports during pandemic-onset remote learning: A national multi-wave daily-diary study. Development and Psychopathology.

[bib44] Watkins N.K., Salafia C., Ohannessian C.M. (2022). Family functioning and anxiety symptoms in adolescents: The moderating role of mindfulness. Journal of Child and Family Studies.

[bib45] Way N., Reddy R., Rhodes J. (2007). Students' perceptions of school climate during the middle school years: Associations with trajectories of psychological and behavioral adjustment. American Journal of Community Psychology.

[bib46] Yoon Y., Eisenstadt M., Lereya S.T., Deighton J. (2023). Gender difference in the change of adolescents' mental health and subjective wellbeing trajectories. European Child & Adolescent Psychiatry.

