# Supplementary material for: Changes in family and school environment during the Covid-19 pandemic and their relationship with changes in psychological distress and loneliness among Norwegian adolescents: The HUNT study
Source: SSM Popul Health. 2025 Feb 21;29:101767. doi: 10.1016/j.ssmph.2025.101767 (PMC11910670; doi:10.1016/j.ssmph.2025.101767)
Supplement: Multimedia component 1 [file mmc1.docx]

**Supplementary document**

Test for measurement invariance

| Table S1. Test for measurement invariance for the univariate LCS models | | | | | | | |
| --- | --- | --- | --- | --- | --- | --- | --- |
| Model | χ² (df) | RMSEA | CFI | TLI | SRMR | BIC | Decision |
| Psychological distress |  |  |  |  |  |  |  |
| Scalar Invariance | 205.21 (37) p<0.001 | 0.054 | 0.970 | 0.963 | 0.035 | 32314.28 | Accepted |
| Residual Invariance | 249.67 (42) p<0.001 | 0.056 | 0.963 | 0.960 | 0.048 | 32338.08 | - |
| Loneliness |  |  |  |  |  |  |  |
| Scalar Invariance | 3.68 (1) p=0.055 | 0.041 | 0.998 | 0.988 | 0.011 | 17234.64 | - |
| Residual Invariance | 4.52 (3) p=0.210 | 0.018 | 0.999 | 0.998 | 0.011 | 17222.17 | Accepted |
| Family cohesion |  |  |  |  |  |  |  |
| Scalar Invariance | 62.55 (9) p<0.001 | 0.062 | 0.980 | 0.967 | 0.032 | 21928.89 | - |
| Partial Metric Invariance* | 25.78 (8) p=0.001 | 0.038 | 0.993 | 0.988 | 0.020 | 21892.97 | - |
| Residual Invariance | 32.56 (11) p<0.001 | 0.035 | 0.992 | 0.989 | 0.025 | 21885.62 | Accepted |
| Teacher support |  |  |  |  |  |  |  |
| Scalar Invariance | 73.17 (21) p<0.001 | 0.040 | 0.987 | 0.983 | 0.021 | 23025.97 | - |
| Residual Invariance | 80.20 (25) p<0.001 | 0.038 | 0.986 | 0.985 | 0.024 | 23009.94 | Accepted |
| Peer support |  |  |  |  |  |  |  |
| Scalar Invariance | 45.08 (9) p<0.001 | 0.051 | 0.982 | 0.970 | 0.022 | 18017.46 |  |
| Residual Invariance | 48.34 (12) p<0.001 | 0.044 | 0.982 | 0.977 | 0.022 | 18001.48 | Accepted |
| * Freed the time invariant factor loading for item Comfortable | | | | | | | |

Final model fits

| Table S2. Unadjusted univariate 2W-LCS models | | | | | |
| --- | --- | --- | --- | --- | --- |
|  | χ² (df) | RMSEA | CFI | TLI | SRMR |
| Psychological distress | 205.21 (37), p<0.001 | 0.054 | 0.970 | 0.963 | 0.035 |
| Loneliness | 4.52 (3), p<0.001 | 0.018 | 0.999 | 0.998 | 0.011 |
| Family cohesion | 32.56 (11), p<0.001 | 0.035 | 0.992 | 0.989 | 0.046 |
| Teacher support | 80.20 (25), p<0.001 | 0.038 | 0.986 | 0.985 | 0.024 |
| Peer support | 48.34 (12), p<0.001 | 0.044 | 0.982 | 0.977 | 0.022 |

| Table S3. Adjusted univariate 2W-LCS models predicting change | | | | | |
| --- | --- | --- | --- | --- | --- |
|  | χ² (df) | RMSEA | CFI | TLI | SRMR |
| Psychological distress | 340.11 (62), p<0.001 | 0.054 | 0.956 | 0.947 | 0.042 |
| Loneliness | 17.88 (10), p<0.001 | 0.023 | 0.996 | 0.993 | 0.019 |
| Family cohesion | 61.01 (24), p<0.001 | 0.032 | 0.988 | 0.984 | 0.028 |
| Teacher support | 141.77 (44), p<0.001 | 0.038 | 0.978 | 0.974 | 0.028 |
| Peer support | 118.77 (25), p<0.001 | 0.050 | 0.958 | 0.945 | 0.030 |
| *Note.* Sex, parental education level, and age were included as covariates for the latent variable at T1.  The latent change score was regressed on sex and parental education level. | | | | | |

| Table S4. Model fit for multivariate 2W-LCS models | | | | | |
| --- | --- | --- | --- | --- | --- |
|  | χ² (df) | RMSEA | CFI | TLI | SRMR |
| Family cohesion and psychological distress | 569.26 (146), p<0.001 | 0.044 | 0.957 | 0.951 | 0.044 |
| Family cohesion and loneliness | 152.14 (58), p<0.001 | 0.033 | 0.982 | 0.977 | 0.034 |
| Teacher support and psychological distress | 643.90 (186), p<0.001 | 0.040 | 0.960 | 0.955 | 0.042 |
| Teacher support and loneliness | 210.84 (86), p<0.001 | 0.031 | 0.981 | 0.978 | 0.030 |
| Peer support and psychological distress | 583.66 (147), p<0.001 | 0.044 | 0.952 | 0.945 | 0.041 |
| Peer support and loneliness | 180.75 (59), p<0.001 | 0.037 | 0.973 | 0.966 | 0.035 |
| *Note.* Sex, parental education level, and age were included as covariates for the latent variable at T1.  Sex was included as a covariate for the latent change score. | | | | | |

| Table S5. Model fit for the multi-group analyses | | | | | |  |
| --- | --- | --- | --- | --- | --- | --- |
|  | χ² (df) | RMSEA | CFI | TLI | SRMR | χ² (df) |
| Family cohesion and psychological distress |  |  |  |  |  |  |
| Constrained | 801.35 (285), *p*<0.001 | 0.049 | 0.948 | 0.944 | 0.059 | 4.85 (1), |
| Unconstrained | 796.40 (284), *p*<0.001 | 0.049 | 0.948 | 0.944 | 0.059 | *p*=0.028 |
| Family cohesion and loneliness |  |  |  |  |  |  |
| Constrained | 275.17 (117), *p*<0.001 | 0.042 | 0.970 | 0.967 | 0.056 | 2.78 (1), |
| Unconstrained | 272.42 (116), *p*<0.001 | 0.042 | 0.971 | 0.967 | 0.056 | *p*=0.095 |
| Teacher support and psychological distress |  |  |  |  |  |  |
| Constrained | 887.21 (363), *p*<0.001 | 0.044 | 0.953 | 0.951 | 0.053 | 2,31 (1), |
| Unconstrained | 884.91 (362), *p*<0.001 | 0.044 | 0.954 | 0.951 | 0.053 | *p*=0.128 |
| Teacher support and loneliness |  |  |  |  |  |  |
| Constrained | 326.93 (171), *p*<0.001 | 0.035 | 0.976 | 0.975 | 0.039 | 0.10 (1), |
| Unconstrained | 326.69 (170), *p*<0.001 | 0.035 | 0.976 | 0.975 | 0.039 | *p*=0.753 |
| Peer support and psychological distress |  |  |  |  |  |  |
| Constrained | 795.14 (286), *p*<0.001 | 0.048 | 0.943 | 0.940 | 0.052 | 2.08 (1), |
| Unconstrained | 793.15 (285), *p*<0.001 | 0.049 | 0.045 | 0.940 | 0.051 | *p*=0.149 |
| Peer support and loneliness |  |  |  |  |  |  |
| Constrained | 267.25 (118), *p*<0.001 | 0.041 | 0.966 | 0.962 | 0.046 | 0.09 (1), |
| Unconstrained | 267.29 (117), *p*<0.001 | 0.041 | 0.966 | 0.962 | 0.046 | *p*=0.762 |
| *Note*. Age was included as a covariate for the latent variable at T1. Sex was included as a covariate for the latent variable at T1 and the latent change score. | | | | | | |
